# Evaluation of the Abbott Real Time HCV genotype II assay for Hepatitis C virus genotyping

**DOI:** 10.12669/pjms.315.7454

**Published:** 2015

**Authors:** Fatma Mutlu Sariguzel, Elife Berk, Selma Gokahmetoglu, Baris Derya Ercal, Ilhami Celik

**Affiliations:** 1 Fatma Mutlu Sariguzel, Department of Microbiology, Kayseri Education and Research Hospital, Kayseri, Turkey; 2 Elife Berk, Department of Microbiology, Erciyes University Medical School, Kayseri, Turkey; 3 Selma Gokahmetoglu, Department of Microbiology, Erciyes University Medical School, Kayseri, Turkey; 4 Baris Derya Ercal, Department of Microbiology, Erciyes University Medical School, Kayseri, Turkey; 5 Ilhami Celik, Department of Infectious Diseases and Clinical Microbiology, Kayseri Education and Research Hospital, Kayseri, Turkey

**Keywords:** HCV genotypes, Abbott RealTime HCV Genotype II assay, Sequencing

## Abstract

**Objective:**

The determination of HCV genotypes and subtypes is very important for the selection of antiviral therapy and epidemiological studies. The aim of this study was to evaluate the performance of Abbott Real Time HCV Genotype II assay in HCV genotyping of HCV infected patients in Kayseri, Turkey.

**Methods:**

One hundred patients with chronic hepatitis C admitted to our hospital were evaluated between June 2012 and December 2012, HCV RNA levels were determined by the COBAS® AmpliPrep/COBAS® TaqMan® 48 HCV test. HCV genotyping was investigated by the Abbott Real Time HCV Genotype II assay. With the exception of genotype 1, subtypes of HCV genotypes could not be determined by Abbott assay. Sequencing analysis was used as the reference method.

**Results:**

Genotypes 1, 2, 3 and 4 were observed in 70, 4, 2 and 24 of the 100 patients, respectively, by two methods. The concordance between the two systems to determine HCV major genotypes was 100%. Of 70 patients with genotype 1, 66 showed infection with subtype 1b and 4 with subtype 1a by Abbott Real Time HCV Genotype II assay. Using sequence analysis, 61 showed infection with subtype 1b and 9 with subtype 1a. In determining of HCV genotype 1 subtypes, the difference between the two methods was not statistically significant (*P*>0.05). HCV genotype 4 and 3 samples were found to be subtype 4d and 3a, respectively, by sequence analysis. There were four patients with genotype 2. Sequence analysis revealed that two of these patients had type 2a and the other two had type 2b.

**Conclusion:**

The Abbott Real Time HCV Genotype II assay yielded results consistent with sequence analysis. However, further optimization of the Abbott Real Time HCV Genotype II assay for subtype identification of HCV is required.

## INTRODUCTION

Hepatitis C virus (HCV) infection is an important infectious disease which can cause chronic liver disease, cirrhosis, and hepatocellular carcinoma. There are seven genotypes and more than 80 subtypes of HCV. The various genotypes and subtypes of HCV are associated with different geographical patterns. HCV genotype 1 is the most frequent genotype in Turkey. The prevalence of genotype 4 in Kayseri, a city in Middle Anatolia in Turkey, is higher than the average national HCV genotype distribution.^1^ In previous studies, it has been shown that genotypes 1 and 4 are more resistant than genotypes 2 and 3 to combination therapy.^2^ HCV genotype determination is very important for selecting antiviral therapy, deciding on treatment duration and monitoring response to treatment. In addition, HCV genotyping is an important and essential tool for epidemiological studies and for tracing the source of infection.^3^-^5^

DNA sequence analysis is the gold standard method for HCV genotyping and is used in most laboratories. Recently, several methods targeting different regions of the HCV genome have been used for genotyping, such as: serologically-based methods, line probe assay, allele-specific PCR, and restriction fragment length polymorphism.^6^-^9^ The Versant HCV Genotype 2.0 assay (INNO-LIPA HCV v.2.0, France) is based on the reverse hybridization of the 5´UTR region and core region.^10^ Pyrosequencing assay is a real-time quantitative sequencing analysis technique based on detection of pyrophosphate released during DNA synthesis.^11^ The Abbott Real Time HCV Genotype II assay (Abbott, USA) targets the 5´UTR region, which is highly conserved among HCV genotypes, and the NS5B gene for efficient discrimination between HCV genotypes 1a and 1b.^12^

The aim of this study was to evaluate the performance of Abbott Real Time HCV Genotype II assay in HCV genotyping of HCV infected patients in Kayseri, a city in Middle Anatolia in Turkey.

## METHODS

### Patients

A total of 100 patients (63 female, 37 male) with chronic hepatitis C admitted to the Kayseri Education and Research Hospital were evaluated between June 2012 and December 2012,. HCV RNA viral loads of patients were recorded. The study protocol and the procedures were approved by the Erciyes University Ethical committee and were in accordance with the Helsinki Declaration of 1975 (approval number: 2012/410).

### Quantification of HCV RNA

HCV RNA levels were determined by commercial real time polymerase chain reaction system, the COBAS® AmpliPrep/COBAS® TaqMan® 48 HCV test (Roche, USA).

### HCV Genotyping

HCV genotyping was investigated by the Abbott Real Time HCV Genotype II assay in HCV positive samples. The Abbott Real Time HCV Genotype II assay (Abbott, USA) was used following the manufacturer’s instructions using the Abbott m24sp and Abbott m2000rt modules. The Abbott Real Time HCV Genotype II assay can only determine genotype 1 subtypes. Subtypes of other genotypes could not be determined.

HCV RNA positive samples were sequenced by the ABI Prism 310 Genetic Analyzer (Applied Biosystems, USA). The HCV genotype was identified using the Basic BLAST software from the National Center for Biotechnology Information site.

### Statistical Analysis

A chi-square test was used to determine differences in genotype 1 subtypes as measured by the Abbott Real Time HCV Genotype II assay and sequence analysis.

## RESULTS

There was 100% concordance between the two systems in determining HCV genotype. Genotypes 1, 2, 3 and 4 were observed in 70, 4, 2 and 24 of the 100 patients, respectively, according to both methods. The HCV RNA viral load range of all patients was 1.39×10^4^- 7.35×10^6^IU/mL.

Of the 70 patients with genotype 1, 66 showed infection with subtype 1b and 4 with subtype 1a according to the Abbott Real Time HCV Genotype II assay. Using sequence analysis, 61 of these patients showed infection with subtype 1b and 9 with subtype 1a. The difference between the two methods (Abbott Real Time HCV Genotype II assay and sequence analysis) in determining of HCV genotype 1 subtypes was not statistically significant (*P*>0.05).

HCV genotypes 4 and 3 were found to be subtype 4d and 3a, respectively, by sequence analysis. There were four patients with genotype 2. According to sequence analysis, two of these patients had subtype 2a and two had subtype 2b. Table-I shows a comparison between Abbott Real Time HCV Genotype II assay and sequence analysis in evaluating the 100 samples.

**Table-I:** Comparison of genotype results for 100 samples by the Abbott Real Time HCV Genotype II assay and sequence analysis.

Genotype	Sequence analysis	Abbott Real Time HCV Genotype II assay	P*
1			
1a	9	4	>0.05
1b	61	66	>0.05
2		4	-
2a	2	-	-
2b	2	-	-
3		2	-
3a	2		-
4		24	-
4d	24	-	-
Total	100	100	

P* : for genotype 1subtypes, there was no statistical difference between the two methods.

## DISCUSSION

HCV genotype should be determined prior to initiation of appropriate treatment. The type of treatment given for chronic HCV depends on the genotype of the virus. Compared to the other genotypes, HCV genotype 1 causes more severe hepatic damage and quicker progression to cirrhosis and hepatocellular carcinoma.^13^,^14^ In addition, genotypes 1 and 4 require prolonged treatment compared to genotypes 2 and 3.^13^,^15^ Patients infected with HCV genotype 1 and 4 require 48 weeks of treatment, while patients with genotype 2 and 3 require 24 weeks of treatment.

In Turkey, the most common genotype is genotype 1b, followed by genotype 1a. Genoypes 2a, 3a, 4, 4c occur at lower rates.^16^-^18^ However, in Kayseri, a city in Middle Anatolia in Turkey, the second most common genotype is 4.^1^,^19^ The phylogenetic analysis showed that HCV genotype 4d samples in this region were in 82 - 95% similarity with genotype 4d strains of different geographical regions. Genotype 4d strains are thought to have come to this region 30 - 75 years ago according to molecular clock analysis.^20^

Direct sequence analysis is a method widely used for genotyping.^7^,^8^ However, there are many disadvantages of sequence analysis. For example, it is expensive, time-consuming, and requires experienced staff.^11^ For this reason, semi-automated systems are widely used. The Abbott Real Time HCV Genotype II assay, based on genotype specific real time PCR, has been widely used to analyze HCV genotypes.^1^,^6^,^12^,^21^,^22^

Earlier studies reported that the patients with subtype 1b have a worse prognosis than patients with subtype 1a. As genotype 1 is the most common genotype in Turkey, as well as in many other countries, subtyping is important. Ciotti et al.^12^ evaluated the performance of the Abbott Real Time HCV Genotype II assay and the Versant HCV Genotype 2.0 assay, and reported that there was good agreement between the two assays. They showed that type concordance was 95.9% (117/122) while subtype level concordance for genotype 1 was 95.6% (43/45). However, a study conducted in 2009 showed that Abbott Real Time HCV Genotype II assay failed to correctly identify the HCV genotype 1 subtype in approximately 10% of samples.^23^ Only genotype 1 subtypes can be determined by the Abbott Real Time HCV Genotype II assay. In our study, genotype 1 was observed in 70 of the 100 patients by both Abbott Real Time HCV Genotype II assay and sequence analysis. The concordance between the two systems in determining HCV genotype 1 was 100%. However, in determining the subtype, the subtype of five samples was misclassified as genotype 1b by the Abbott Real Time HCV Genotype II assay. These five samples were classified as genotype 1a by sequence analysis. This difference is not statistically significant.

Sohn et al.^6^ evaluated performance of restriction fragment mass polymorphism (RFMP) and Abbott Real Time HCV Genotype II assay in 66 HCV positive sample. Thirty-nine samples were identified as genotype 1, 22 as genotype 2 and five as genotype 3 by the RFMP method. These results were usually compatible with the Abbott Real Time HCV Genotype II assay, except in three samples. Two samples were identified as mixed genotype (1b+3) by Abbott Real Time HCV Genotype II assay, and as genotype 1 by RFMP. One sample was detected as mixed genotype by Abbott Real Time HCV Genotype II assay, and as genotype 2 by RFMP. Also, their study reported that the subtype results of 15 HCV genotype 1 samples were not compatible between the two methods. In our study, we did not find any mixed genotypes by the Abbott Real Time HCV Genotype II assay. Genotype 2, genotype 3 and 4 results were consistent between the two methods.

Yang et al.^21^ showed that Abbott Real Time HCV Genotype II assay and Versant HCV genotype 2.0 assay have limitations in identifying HCV genotype 6. However, in our study we did not find any genotype 6 by either Abbott Real Time HCV Genotype II assay or sequence analysis.

### Limitations of the study

There are some limitations in our study, such as limited sample size and lack of mixed genotypes.

## CONCLUSION

Concordance between the Abbott Real Time HCV genotype II assay and sequence analysis for determining of major HCV genotypes was good in this study. Although the Abbott Real Time HCV genotype II assay has the additional advantages of automation, rapidity and low labour intensity, we suggest that this system needs improvement for subtype identification of HCV.
